# Supplementary figures and images for: Polyp expansion of passive suspension feeders: a red coral case study
Source: PeerJ. 2019 Jul 9;7:e7076. doi: 10.7717/peerj.7076 (PMC6625502; doi:10.7717/peerj.7076)

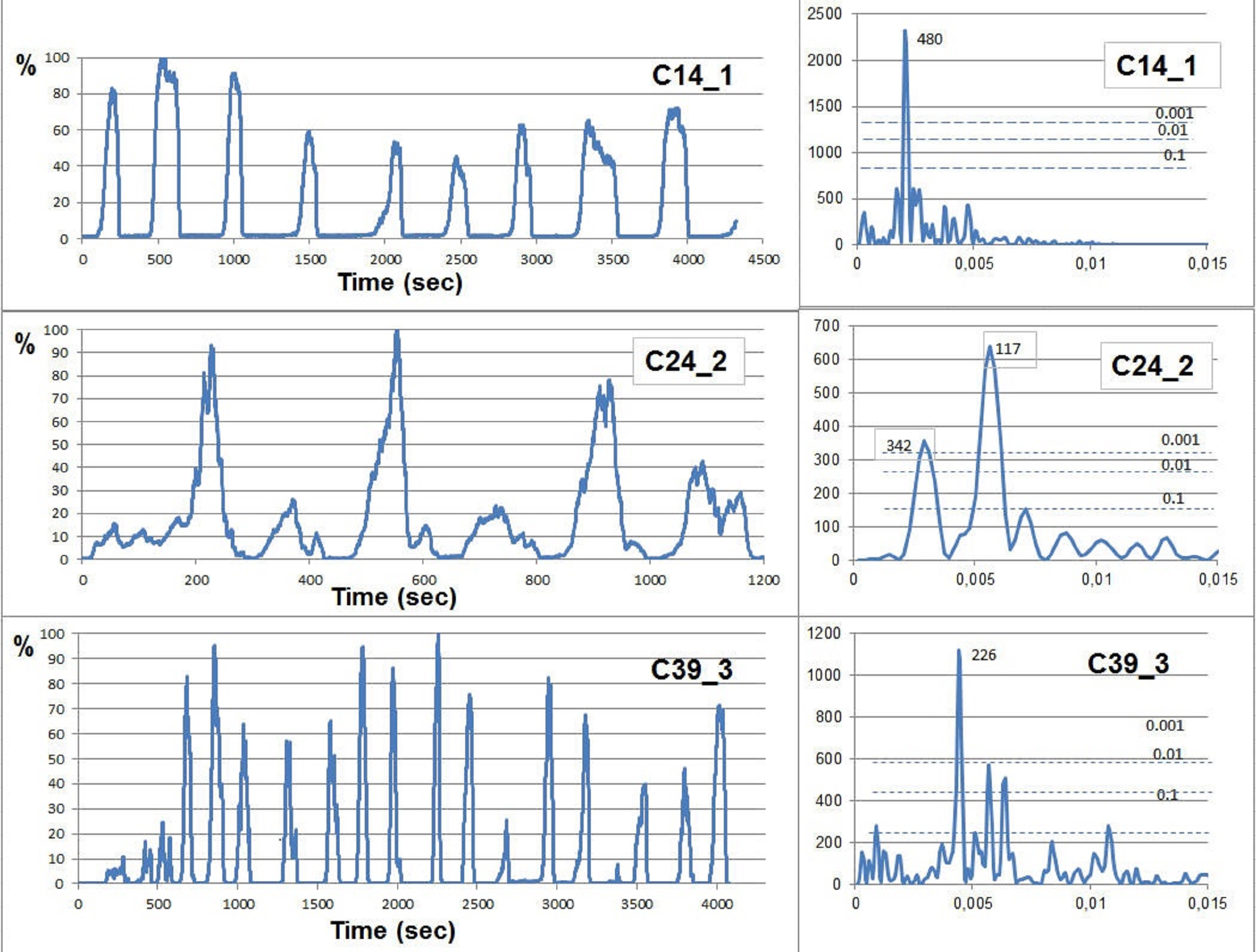

Supplement: Figure S1 — Example of three periodograms from three different colonies (peaks represent polyp expansion), showing endogenous rhythms at 18 °C and still-water conditions. On the left the recorded normalised activities (i.e., the number of pixels divided by the maximum polyp expansion for that experiment); on the right the Lomb periodogram with frequencies on the X axis and number of occurrences on the Y axis. Figures close to the peaks indicate the periods. The 3 dashed lines represent the significance of the peaks, 0.1, 0.01 and 0.001, the smallest value corresponding to the highest significance. [file peerj-07-7076-s001.png]

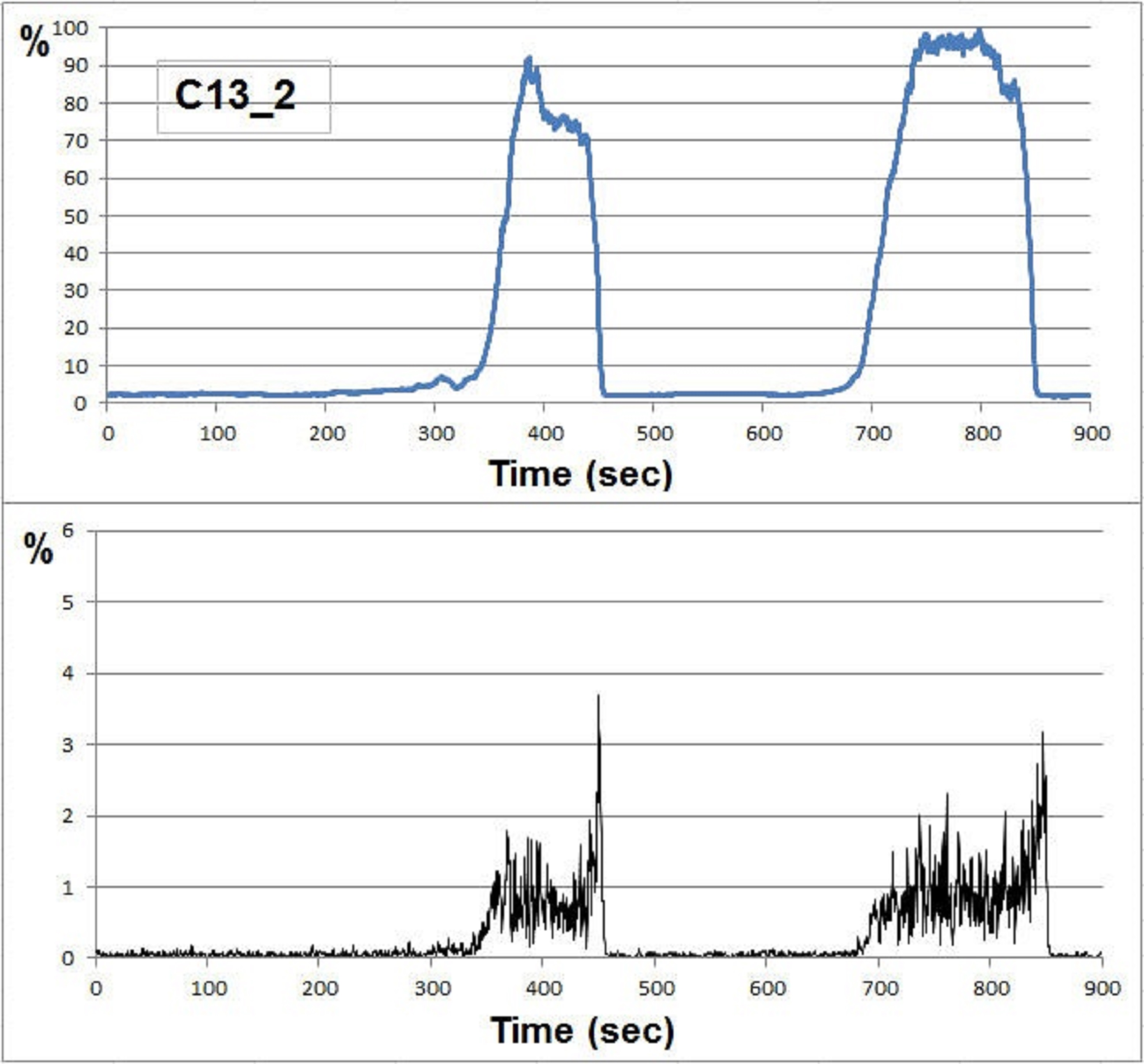

Supplement: Figure S2 — (A) The area below the peaks for a given experiment. (B) The derivative of this curve with absolute values (increase or decrease in polyp expansion). These records usually show a steeper descent after opening. [file peerj-07-7076-s002.png]
