# Supplementary material for: Polyp expansion of passive suspension feeders: a red coral case study
Source: PeerJ. 2019 Jul 9;7:e7076. doi: 10.7717/peerj.7076 (PMC6625502; doi:10.7717/peerj.7076)
Supplement: Supplemental Information 4 — Tables S1–S8: tables with the different statistical analysis. [file peerj-07-7076-s004.docx]

**Polyp expansion of passive suspension feeders: a red coral case study**

Sergio Rossi^1,2^, Lucia Rizzo^3^, Jean-Claude Duchêne^4^

^1^ DiSTeBA, Campus Ecotekne, University of Salento, 73100 Lecce, Italy

^2^ Institute of Environmental and Technology (ICTA), Universitat Autònoma de Barcelona (UAB), Edifici Z, Passeig de les Columnes, Universitat Autònoma de Barcelona, Cerdanyola del Vallés 08193, Spain

^3^ Stazione Zoologica Anton Dohrn, Napoli, Italy

^4^ Station Marine d’Arcachon, Environnements et Paleoenvironnements Océaniques, UMR 5805 (Université Bordeaux 1-CNRS), 2 rue du Professeur Jolyet, 33120 Arcachon, France

Corresponding author: [sergio.rossi@unisalento.it](mailto:sergio.rossi@unisalento.it)

**Table S1.** Results of multivariate permutational analyses (PERMANOVA) of variation in *C. rubrum* polyp expansion and seston composition among cycles (Cy) and sampling times (Ti(Cy)). df = degree of freedom; MS = mean sum of squares; F = F value by permutation. *** = P< 0.001.

|  |  |  | Polyp Expansion | | |  | Seston composition | | |
| --- | --- | --- | --- | --- | --- | --- | --- | --- | --- |
| Source | df |  | MS | Pseudo-F | P(perm) |  | MS | Pseudo-F | P(perm) |
| Cy | 4 |  | 92.929 | 18.296 |  |  | 32.743 | 16.945 |  |
| Ti(Cy) | 14 |  | 81.599 | 16.065 | *** |  | 13.4 | 6.9347 | *** |
| Res | 38 |  | 5.0792 |  |  |  | 1.9323 |  |  |
| Total | 56 |  |  |  |  |  |  |  |  |

**Table S2.** Results of pairwise tests contrasting different factor levels. df = degree of freedom; MS = mean sum of squares; T = T value; P = probability level. ns = not significant; * = P<0.05; ** = P < 0.01; *** = P< 0.001.

|  | t | P(MC) |  | T | P(MC) |  | t | P(MC) |  | t | P(MC) |  | t | P(MC) |
| --- | --- | --- | --- | --- | --- | --- | --- | --- | --- | --- | --- | --- | --- | --- |
|  | C1 | |  | C2 | |  | C3 | |  | C4 | |  | C5 |  |
|  | **Polyp Expansion** | | | | | | | | | | | | | |
| T1 vs T2 | 5.3712 | ** |  | 1.4124 | ns |  | 5.1708 | ** |  | 1 | ns |  | 9.9367 | *** |
| T1 vs T3 | 2.5328 | * |  | 2.3306 | ns |  |  |  |  | 1.5695 | ns |  | 10.332 | *** |
| T1 vs T4 | 2.9011 | * |  | 3.0772 | * |  | 3.0102 | * |  | 6.2224 | ** |  | 0.88548 | ns |
| T2 vsT3 | 90.254 | *** |  | 0.53199 | ns |  |  |  |  | 1.642 | ns |  | 1 | ns |
| T2 vsT4 | 6.9985 | ** |  | 4.1082 | ** |  | 1.6991 | ns |  | 6.3454 | ** |  | 3.7026 | * |
| T3 vs T4 | 6.6688 | ** |  | 10.119 | *** |  |  |  |  | 3.7404 | ** |  | 3.7495 | * |
|  | **Seston Composition** | | | | | | | | | | | | | |
| T1 vs T2 | 5.9206 | ** |  | 2.0901 | ns |  | 2.3717 | * |  | 2.3458 | ns |  | 1.9988 | ns |
| T1 vs T3 | 3.3612 | ** |  | 1.6031 | ns |  |  |  |  | 2.4643 | * |  | 3.0539 | * |
| T1 vs T4 | 4.2089 | ** |  | 1.7729 | ns |  | 2.0148 | ns |  | 2.2335 | * |  | 3.1312 | * |
| T2 vsT3 | 4.8647 | ** |  | 2.5371 | * |  |  |  |  | 2.8007 | * |  | 2.661 | * |
| T2 vsT4 | 4.5115 | ** |  | 1.9588 | ns |  | 1.6917 | ns |  | 1.5277 | ns |  | 2.561 | * |
| T3 vs T4 | 2.7498 | * |  | 1.0386 | ns |  |  |  |  | 2.7262 | * |  | 3.0224 | * |

**Table S4.** Results of multivariate permutational analyses (PERMANOVA) of variation in the zooplankton community among cycles (Cy). df = degree of freedom; MS = mean sum of squares; Pseudo-F = F value by permutation. *** = P< 0.001.

|  |  | Zooplankton community | | |
| --- | --- | --- | --- | --- |
| Source | df | MS | Pseudo-F | P(perm) |
| Cy | 4 | 703.58 | 3.9485 | *** |
| Res | 14 | 178.19 |  |  |
| Total | 18 |  |  |  |

**Table S4.** Results of SIMPER analysis showing the percentage contribution of the species to the dissimilarity among cycles.

|  |  |  |  |  |  | C1 vs C2 | | C1 vs C3 | | C2 vs C3 | | C1 vs C4 | | C2 vs C4 | | C3 vs C4 | | C1 vs C5 | | C2 vs C5 | | C3 vs C5 | | C4 vs C5 | |
| --- | --- | --- | --- | --- | --- | --- | --- | --- | --- | --- | --- | --- | --- | --- | --- | --- | --- | --- | --- | --- | --- | --- | --- | --- | --- |
|  | C1 | C2 | C3 | C4 | C5 | Dis. = 18,03 | | Dis. = 25,09 | | Dis. = 22,09 | | Dis. = 27,02 | | Dis. = 25,06 | | Dis. = 18,32 | | Dis. = 30,74 | | Dis. = 30,12 | | Dis. = 21,31 | | Dis. = 18,85 | |
| Taxa | Av.Ab | Av.Ab | Av.Ab | Av.Ab | Av.Ab | Av.Dis | % | Av.Dis | % | Av.Dis | % | Av.Dis | % | Av.Dis | % | Av.Dis | % | Av.Dis | % | Av.Dis | % | Av.Dis | % | Av.Dis | % |
| Crustacea | 0,75 | 1,42 | 1,21 | 1,2 | 0,38 | 1,44 | 8,02 | 1,33 | 5,28 | 0,98 | 4,44 | 1,46 | 5,39 | 1,39 | 5,55 | 1,36 | 7,41 | 0,93 | 3,04 | 1,26 | 4,19 | 1,11 | 5,21 | 1,14 | 6,06 |
| Gastropoda | 0,75 | 1,43 | 1,51 | 1,87 | 1,41 | 1,18 | 6,53 | 1,17 | 4,67 | 0,42 | 1,91 | 1,33 | 4,93 | 0,51 | 2,02 | 0,45 | 2,48 | 1,27 | 4,14 | 0,88 | 2,93 | 0,8 | 3,74 | 0,73 | 3,88 |
| Syphonophora | 1,11 | 0,65 | 1,72 | 1,91 | 2,15 | 1,18 | 6,53 | 0,85 | 3,37 | 1,4 | 6,35 | 0,95 | 3,53 | 1,48 | 5,89 | 1,11 | 6,03 | 1,15 | 3,76 | 1,69 | 5,61 | 0,55 | 2,58 | 0,47 | 2,5 |
| L.Invertebr. | 1,08 | 1,19 | 1,79 | 1,49 | 2,16 | 1,02 | 5,67 | 0,96 | 3,81 | 0,78 | 3,54 | 0,66 | 2,42 | 0,56 | 2,23 | 1,3 | 7,08 | 1,31 | 4,25 | 1,2 | 3,99 | 0,64 | 3,01 | 0,71 | 3,74 |
| Harpacticoida | 3,41 | 3,16 | 3,28 | 3,04 | 3,21 | 0,97 | 5,35 | 0,78 | 3,09 | 0,47 | 2,12 | 0,79 | 2,91 | 0,45 | 1,8 | 0,36 | 1,98 | 0,79 | 2,57 | 0,58 | 1,92 | 0,52 | 2,43 | 0,5 | 2,66 |
| Misidiacea | 0,64 | 0 | 0 | 0,7 | 0,73 | 0,96 | 5,3 | 0,85 | 3,38 | 0 | 0 | 1,18 | 4,37 | 0,78 | 3,13 | 0,9 | 4,93 | 0,86 | 2,8 | 0,82 | 2,73 | 0,75 | 3,53 | 1,01 | 5,35 |
| Amphipoda | 0,62 | 0,27 | 0 | 0 | 0 | 0,88 | 4,86 | 0,77 | 3,08 | 0,34 | 1,53 | 0,7 | 2,59 | 0,31 | 1,22 | 0 | 0 | 0,69 | 2,24 | 0,3 | 1 | 0 | 0 | 0 | 0 |
| Salpae | 1,45 | 0,98 | 2,42 | 3,17 | 2,99 | 0,82 | 4,55 | 1,24 | 4,94 | 1,86 | 8,44 | 1,98 | 7,34 | 2,55 | 10,15 | 0,4 | 2,19 | 1,68 | 5,47 | 2,24 | 7,42 | 0,88 | 4,12 | 0,83 | 4,41 |
| Lamellibranchia | 1 | 1,41 | 1,18 | 1,76 | 2,16 | 0,8 | 4,45 | 1,11 | 4,42 | 1,01 | 4,59 | 0,93 | 3,45 | 0,47 | 1,86 | 0,88 | 4,81 | 1,33 | 4,31 | 0,87 | 2,87 | 1,16 | 5,42 | 0,53 | 2,83 |
| Ostracoda | 1,11 | 1,41 | 0,59 | 1,48 | 1,43 | 0,78 | 4,32 | 1,23 | 4,9 | 1,38 | 6,27 | 0,62 | 2,28 | 0,13 | 0,53 | 1,01 | 5,52 | 1,02 | 3,33 | 0,78 | 2,58 | 1,24 | 5,81 | 0,62 | 3,31 |
| L.Misis-Zoe | 0,29 | 0,38 | 0,47 | 1,66 | 0,92 | 0,73 | 4,06 | 0,71 | 2,83 | 0,78 | 3,52 | 1,61 | 5,98 | 1,53 | 6,12 | 0,9 | 4,92 | 0,99 | 3,21 | 1 | 3,31 | 0,92 | 4,32 | 0,97 | 5,16 |
| Eggs | 1,92 | 2,18 | 1,95 | 2,23 | 2,22 | 0,7 | 3,87 | 0,54 | 2,17 | 0,44 | 1,98 | 0,61 | 2,25 | 0,29 | 1,16 | 0,4 | 2,16 | 0,61 | 1,99 | 0,47 | 1,57 | 0,47 | 2,2 | 0,41 | 2,17 |
| Protozoa | 0,49 | 0 | 0 | 1,95 | 2,34 | 0,69 | 3,82 | 0,62 | 2,46 | 0 | 0 | 1,77 | 6,56 | 2,26 | 9,03 | 0,84 | 4,57 | 2,14 | 6,95 | 2,69 | 8,92 | 2,45 | 11,49 | 0,41 | 2,16 |
| Medusae | 0,28 | 0,3 | 1,68 | 1,65 | 1,9 | 0,63 | 3,5 | 1,8 | 7,19 | 1,77 | 8,03 | 1,73 | 6,42 | 1,72 | 6,87 | 0,72 | 3,91 | 1,78 | 5,8 | 1,77 | 5,87 | 0,73 | 3,43 | 1,05 | 5,57 |
| Cyclopoida | 4,1 | 3,79 | 5,44 | 5,61 | 6,35 | 0,63 | 3,49 | 1,71 | 6,82 | 2,09 | 9,46 | 1,7 | 6,3 | 2,03 | 8,09 | 1,55 | 8,45 | 2,64 | 8,6 | 2,88 | 9,57 | 1,79 | 8,4 | 2,12 | 11,27 |
| Calanoida | 2,81 | 3,08 | 4,37 | 4,21 | 4,84 | 0,62 | 3,46 | 1,97 | 7,87 | 1,64 | 7,42 | 1,6 | 5,92 | 1,29 | 5,17 | 0,6 | 3,29 | 2,27 | 7,38 | 1,99 | 6,61 | 1,36 | 6,36 | 1,33 | 7,04 |
| Nauplii | 3,22 | 3,36 | 4,16 | 3,97 | 4,71 | 0,62 | 3,44 | 1,27 | 5,04 | 1,04 | 4,72 | 1,03 | 3,8 | 0,89 | 3,55 | 1,17 | 6,36 | 1,66 | 5,41 | 1,47 | 4,87 | 0,85 | 3,99 | 1,07 | 5,67 |
| Tintinida | 1,7 | 1,48 | 2,12 | 3,08 | 2,75 | 0,6 | 3,34 | 0,85 | 3,38 | 0,83 | 3,78 | 1,59 | 5,87 | 1,82 | 7,28 | 0 | 0 | 1,22 | 3,97 | 1,39 | 4,63 | 0,85 | 4 | 0,56 | 2,96 |
| Isopoda | 0,27 | 0,27 | 0 | 0 | 0,27 | 0,57 | 3,17 | 0,34 | 1,35 | 0,34 | 1,53 | 0,31 | 1,13 | 0,31 | 1,22 | 0,31 | 1,7 | 0,48 | 1,56 | 0,48 | 1,59 | 0,31 | 1,47 | 0,29 | 1,52 |
| Polichaeta | 1,4 | 1,74 | 2,2 | 2,06 | 2,5 | 0,55 | 3,06 | 1,03 | 4,1 | 0,61 | 2,77 | 0,76 | 2,83 | 0,44 | 1,74 | 2,06 | 11,26 | 1,26 | 4,11 | 0,87 | 2,89 | 0,32 | 1,52 | 0,43 | 2,29 |
| Others | 1,16 | 0,89 | 0,4 | 1,16 | 0,4 | 0,52 | 2,89 | 0,99 | 3,95 | 0,89 | 4,03 | 0,65 | 2,42 | 0,82 | 3,28 | 0,25 | 1,38 | 1,1 | 3,56 | 0,96 | 3,19 | 0,66 | 3,1 | 0,98 | 5,21 |
| Apendicularia | 2,38 | 2,35 | 3,19 | 3,33 | 3,5 | 0,51 | 2,81 | 1,04 | 4,13 | 1,08 | 4,9 | 1,08 | 3,98 | 1,12 | 4,46 | 0,4 | 2,19 | 1,37 | 4,46 | 1,38 | 4,59 | 0,83 | 3,88 | 0,77 | 4,1 |
| Cladocera | 1,58 | 1,61 | 2,36 | 2,67 | 2,79 | 0,38 | 2,11 | 1,01 | 4,02 | 0,96 | 4,36 | 1,26 | 4,65 | 1,21 | 4,85 | 0,43 | 2,36 | 1,45 | 4,73 | 1,45 | 4,81 | 1,17 | 5,47 | 1,1 | 5,81 |
| Echinodermata | 1,41 | 1,38 | 1,21 | 1,31 | 1,24 | 0,25 | 1,41 | 0,94 | 3,74 | 0,95 | 4,3 | 0,72 | 2,68 | 0,7 | 2,79 | 0,92 | 5,02 | 0,73 | 2,36 | 0,7 | 2,31 | 0,96 | 4,5 | 0,82 | 4,33 |

**Table S5.** Results of the multivariate permutational analyses (PERMANOVA) of variation in *C. rubrum* polyp expansion under different temperature (Te) and current (Cu) conditions. df = degree of freedom; MS = mean sum of squares; F = F value by permutation. * = P < 0.01; *** = P< 0.001.

| Source | Df | MS | Pseudo-F | P(perm) |
| --- | --- | --- | --- | --- |
| Te | 2 | 1.57E+05 | 3.5749 |  |
| Cu | 2 | 9.34E+05 | 21.251 |  |
| Te x Cu | 4 | 2.55E+05 | 5.7999 | *** |
| Res | 99 | 43973 |  |  |
| Total | 107 |  |  |  |

**Table S6.** Results of pairwise tests contrasting different levels of the two factors tested. C0= still water; C1= 3 cm s^-1^; C2= 6 cm s^-1^. T1= 13° C; T2=18° C; T3= 25° C. df = degree of freedom; MS = mean sum of squares; T = T value; P(MC) = probability level after Monte Carlo simulations. ns = not significant; * = P<0.05; ** = P < 0.01; *** = P< 0.001.

| Groups | T | P(perm) | t | P(perm) | t | P(perm) |
| --- | --- | --- | --- | --- | --- | --- |
|  | C0 | | C1 | | C2 | |
| T1 vs T2 | 1.0294 | ns | 3.6746 | ** | 2.2583 | * |
| T1 vs T3 | 1.4163 | ns | 1.758 | ns | 5.1054 | *** |
| T2 vs T3 | 0.86483 | ns | 3.7428 | *** | 0.72957 | ns |

**Table S7.** Results of the multivariate permutational analyses (PERMANOVA) of variation in the polyp expansion of *C. rubrum* exposed to different nutritional stimuli (Nu). df = degree of freedom; MS = mean sum of squares; F = F value by permutation. *** = P< 0.001.

| Source | df | MS | Pseudo-F | P(perm) |
| --- | --- | --- | --- | --- |
| Nu | 3 | 9.7956 | 17.149 | *** |
| Res | 28 | 0.5712 |  |  |
| Total | 31 |  |  |  |

**Table S8.** Results of the pairwise tests contrasting the different temperatures and currents tested in the laboratory experiments. N0= no stimulus; N1= 40 ml; N2= 120 ml; N3=zooplankton (aprox 1500 ind m^-3^). df = degree of freedom; MS = mean sum of squares; T = T value; P(MC) = probability level after Monte Carlo simulations. ns = not significant; ** = P < 0.01; *** = P< 0.001.

| Groups | t | P(MC) |
| --- | --- | --- |
| N0 vs N1 | 2.9936 | ** |
| N0 vs N2 | 4.5724 | *** |
| N0 vs N3 | 5.5413 | *** |
| N1 vs N2 | 1.948 | ns |
| N1 vs N3 | 3.5632 | ** |
| N2 vs N3 | 3.97 | ** |

SUPPLEMENTARY ELECTRONIC MATERIAL FIGURE Captions

**Figure S1 SEM: Periodograms from three different colonies.** Example of three periodograms from three different colonies (peaks represent polyp expansion), showing endogenous rhythms at 18°C and still-water conditions. On the left the recorded normalised activities (i.e. the number of pixels divided by the maximum polyp expansion for that experiment); on the right the Lomb periodogram with frequencies on the X axis and number of occurrences on the Y axis. Figures close to the peaks indicate the periods. The 3 dashed lines represent the significance of the peaks, 0.1, 0.01 and 0.001, the smallest value corresponding to the highest significance.

**Figure S2 SEM: Records of individual polyp activity.** (A) The area below the peaks for a given experiment. (B) The derivative of this curve with absolute values (increase or decrease in polyp expansion). These records usually show a steeper descent after opening.

VIDEO RECORDING: *Corallium rubrum* polyp activity at 18°C and 3 cm s^-1^ current speed.
